# The “Economic Battle” Now and Then: (E)valuation Patterns of Distributive Justice in Cuban State-Socialism

**DOI:** 10.1007/s11211-021-00372-1

**Published:** 2021-07-01

**Authors:** Nina Jany

**Affiliations:** Department of Social Work, Social Policy and Global Development, University of Fribourg, Fribourg, Switzerland

**Keywords:** Distributive justice, Multiprinciple approach, (E)valuation patterns, Ideology, State-socialism, Cuba

## Abstract

This article disentangles and explores some commonly made assumptions about *egalitarian state-socialist ideologies*. Based on the conceptual framework of the multiprinciple approach of justice, it presents the results of an in-depth analysis of (e)valuation patterns of distributive justice in Cuban state-socialism. The analysis mainly focuses on ideational conceptions of distributive justice *(just rewards)*, but it also accounts for distribution outcomes and resulting (in)equalities *(actual rewards)*. The results of the comparative case study of the Cuban framework of institutions and political leaders’ views in two periods of time, the early 1960s and the 2010s, point to (e)valuation patterns that are generally labelled as *egalitarian*, such as the allocation rules of outcome equality and (non-functional) needs. However, contrary to common assumptions about egalitarian state-socialist ideologies, the results also point to several other patterns*,* including equity rules as well as functional and productivist allocation rules. I argue that many of these (e)valuation patterns, in their connection to the discursive storyline of the Cuban *economic battle,* are indeed compatible with *egalitarian state-socialist ideology.*

## Introduction

With the fall of the Berlin wall by the end of the twentieth century, the sociology of justice has seen a rise in studies in the field of comparative attitude research (Liebig & Sauer, 2016). Many of these studies that examine differences in attitudes towards income inequalities, occupational hierarchies, and redistribution include a “Communist variable” (Kelley & Evans, 1993) to test for “the role of Communism” (Alesina & Fuchs-Schündelin, 2007) or “the influence of communist history” (Kulin & Meuleman, 2015). Roughly speaking, hypotheses linked to such variables assume that differences in individual attitudes towards distributive justice can be attributed to specific characteristics of (former) (state-)socialist and (market-)capitalist societies, respectively. This often includes the assumption that in countries that formerly belonged to the so-called communist bloc*,* people are critical towards large inequalities because of a “dominant ideology of equality (i.e. egalitarian, non-functionalistic, non-meritocratic views)” (Hadler, 2005, p. 136). We find similar considerations in everyday conversations, mass media, and in academic reflections on social justice, also regarding (allegedly) opposite ideologies in (market-)capitalist societies. Schumann (2001), for example, identifies the allocation rule of contribution and preferences for economic incentives (reflected, e.g. in pay-for-performance systems) as proprietary characteristics of a capitalist approach to distributive justice. In the socialist approach, in turn, “incentives are thought to be corrosive to the well-being of members of society. Instead, the socialist points to the importance of abilities and needs” (Schumann, 2001, p. 102).

Culturalist approaches such as *(dominant) ideology theory* (e.g. Abercrombie & Turner, 1978; Kluegel & Smith, 1986) presume, explicitly or implicitly, a common societal or cultural heritage in the form of values, beliefs, and attitudes. Generally, this presupposes the existence of a more or less homogenous framework of institutions and political leaders’ and/or elites’ views, which influence individual attitudes. However, in comparative attitude research, descriptions of such frameworks are often limited to short references regarding, for instance, destratification policies favouring blue-collar workers (e.g. Gijsberts, 2002) or low wage spreads (e.g. Alesina & Fuchs-Schündelin, 2007) in (former) state-socialist countries. Kelley & Zagorski (2005, p. 352) speak of **“**the ideological egalitarianism of Communism, its sustained propaganda for equality, and very low levels of actual inequality in Communist society”. Ignácz (2018, p. 4) states that “most socialist regimes kept income inequality to a relatively low level” and that “regimes typically communicated egalitarian ideology”. As a result, the dissolution of the Soviet Union and the transition processes in these societies are presumed to have caused *shifts* in these ideologies, leading researchers to suppose, for example, that “the renunciation of the official communist egalitarian ideology will have induced a shift towards an ideology of rewards for achievement” (Gijsberts, 2002, p. 282).

Empirical results (e.g. Evans & Kelley, 2018; Kelley & Zagorski, 2005; Kreidl, 2000) have questioned the ability of culturalist approaches such as (dominant) ideology theory to explain individual justice evaluations. As Hadler (2005, p. 135) points out, “one doubt [regarding the ideology thesis] concerns the statement that elites are able to convey their views to the population”. However, the assumption that elites’ views and institutional frameworks in state-socialist contexts were or are of a predominantly egalitarian, non-functionalistic and non-meritocratic character seems to be less discussed. This is why, in this article, I propose to explore this assumption, notably by using the example of the Cuban framework of institutions and political leaders’ views.

Interestingly, debates on Cuban state-socialist ideology are characterized by conjectures similar to the above-discussed. The Caribbean island has, until today, formally remained a constitutionally socialist state with a mainly state-led and centrally planned economy. Nevertheless, the dissolution of the Soviet Union and the deep economic crisis that subsequently plagued Cuba are often depicted as an important turning point regarding both actual distribution and ideational conceptions of distributive justice in the country. The crisis of the 1990s is said to be the origin of important changes in socio-economic stratification, characterized in particular by growing inequalities. At the same time, observers notice changing directions in the Cuban government’s “post-Soviet economic policies that challenged Cuba's egalitarian project” (Gordy, 2015, pp. 5–6). Especially the most recent and ongoing reform process, the so-called *update of the socio-economic model*, which also coincided with changes in political leadership (most importantly the presidential changes from Fidel to Raúl Castro in 2008, and from Raúl Castro to Miguel Díaz-Canel in 2018), is said to be accompanied by a “new understanding of socialism” (Ritter & Henken, 2015, p. 2). Many observers contend that, although *social justice* still seems to be an important key word of Havana’s official revolutionary rhetoric, its ideational content may have changed over the past decades.

Among frequently mentioned illustrations for this are the repeated critiques of the Cuban state wage system, voiced not only by the general public but also by the government (that has adopted several wage reforms in recent years). According to Domínguez (2017, p. 2), the political course initiated by former president Raúl Castro and its accompanying rhetoric also suggest that “something is new and different with regard to social policies: their aim is to provide Cuba with “an efficient and sustainable system,” not merely to promote justice in our time”. In summary, observers contend that there is a growing ideational emphasis on (economic) performance criteria, material progress, and efficiency in Cuban discourses and policies (e.g. Bye, 2020; Gómez, 2014; Hoffmann, 2016). Such observations are then often contrasted with the perception of a more egalitarian design and rhetorical framing of social and labour policies as they were set out at the beginning of the Cuban revolution^1^ in the early 1960s. Social development, the (alleged) previous focus of the government, would now, in contemporary Cuba, be merely conceived of as “a ‘derivative’ of economic development” (Echevarría et al., 2019, p. 153, my translation).

However, parts of the literature on the history of the Cuban revolution suggest that changing emphases of policy approaches and political ideology could be observed much earlier than at the end of the twentieth century. According to this viewpoint, Cuba has, since the early days of the revolution in the 1960s, “experimented with multiple models of economic organization and development strategies” (Mesa-Lago, 1994, p. 9, my translation). This included “seesaws in economic and labor policy” (Farber, 2011, p. 140) which, in turn, have not been framed by one uniform, but by various and sometimes even rival conceptions of distributive justice and allocation rules. In this article, I follow-up on this viewpoint by linking it to the multiprinciple approach of justice. This approach assumes the existence—and possible co-existence—of different allocation rules and contends that such rules can be interpreted differently in different (cultural) contexts, i.e. be further specified in various sub-rules, as will be explained in the next section.

With the exploration of (e)valuations of distributive justice in Cuban institutions and statements of political leaders, I intend to contribute to social justice research by applying and extending already existing “maps”—as Bank (2016) calls it—of (e)valuation patterns of distributive justice to the Cuban case. This hopefully allows to disentangle, elaborate, or correct some of the above-discussed assumptions in both international and Cuban-specific research.

## Conceptual Considerations

The assumption that (former) state-socialist societies were or are characterized by an *egalitarian ideology* generally refers to different conceptual levels. In the comparative attitude studies discussed in the introduction, egalitarian individual ideologies are assumed to be influenced by a corresponding egalitarian framework of institutions and political leaders’ views. Regarding the latter, the studies in question often refer to both ideational conceptions of distributive justice in this framework (e.g. by using keywords such as *regime* or *state ideology*) and actual outcomes of distribution (which, in state-socialist societies, are generally assumed to be characterized by low levels of income inequality, low wage spreads and greater rewards for blue-collar workers). To examine Cuban state-socialist ideology, I will thus concentrate, in the following, on (e)valuation patterns of distributive justice in both institutions and political leaders’ views. I use the term *(e)valuation patterns* following Lamont’s (2012, p. 208) considerations that “valuation practices (giving worth or value) and evaluative practices (assessing—how an entity attains a certain type of worth)” consist of different sub-processes of categorization and legitimation. The theoretical distinction between *just rewards* and *actual rewards* provided by Jasso et al. (2016) allows to separately approach the levels of ideational conceptions of distributive justice on the one hand, and distribution outcomes and resulting (in)equalities on the other.

As also discussed in the introduction, the assumption of *egalitarian state-socialist ideology* is often linked to several additional conjectures. Firstly, assuming the dominance of egalitarian (e)valuation patterns in institutions and political leaders’ views generally implies the conjecture that these are characterized first and foremost by the allocation rules of outcome equality and/or need. Secondly, it is often explicitly or implicitly assumed that *egalitarian ideology* is contrary to or incompatible with the allocation rule of equity (also referred to as contribution-, merit-, achievement-, or proportionality rule). Thirdly, we have seen that state-socialist *egalitarianism* and its underlying allocation rules are often perceived to be purely normative or non-functionalistic (e)valuation patterns. This means that an allocation rule, for instance, is presented as an “intrinsically valued goal”, i.e. as an end in itself and not as “a strategy for the attainment of other ends” (Törnblom & Kazemi, 2015, p. 22, referring to Van Avermaet et al., 1978).^2^ Fourthly, societal changes in the framework of transformation processes—such as those that took place after the fall of the Berlin wall—are assumed to be accompanied by changes in (e)valuation patterns of distributive justice. This is often expressed in the conjecture that, broadly speaking, transformation processes in former state-socialist societies are accompanied by a transition from *egalitarian* to *equity* logic. In the debates on Cuban transformation processes we also find the conjecture of an increasing incidence of *economistic* or *productivist* (e)valuation patterns.

**Fig. 1:**
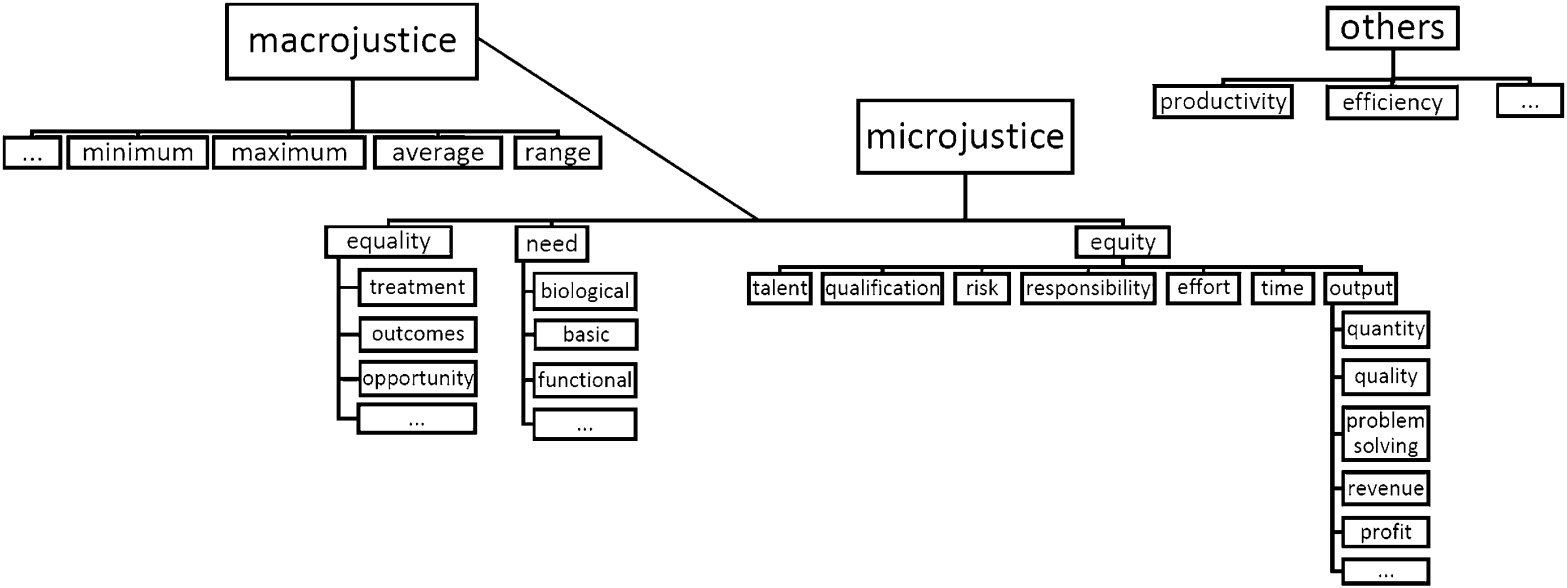
Allocation rules

In order to examine these assumptions more closely with respect to the Cuban case, I rely on insights and concepts developed in the framework of the multiprinciple approach of distributive justice (also referred to as the contingency or classification approach, see Törnblom & Kazemi, 2015, p. 25). Since a wide range of scholars has contributed to the development and evolution of this approach, I refer, in the following, to the comprehensive overview of concepts and former empirical results provided by Törnblom and Kazemi (2015). I use the basic conceptual distinction between the allocation rules of equity, equality, and need to approach (e)valuation patterns of distributive justice in the Cuban framework of institutions and political leaders’ views. Hereby, I consider that these three principles can take different shapes or configurations and that they can be further differentiated into various sub-rules*.* Additionally, I account for other allocation rules, which are not considered to be distributive justice rules per se but are linked to issues of distributive justice. Moreover, especially when it comes to discussing so-called *state ideologies*, it seems helpful to distinguish between “microjustice (the fairness of rewards to individual recipients) and macrojustice (the aggregate fairness of reward in a society)” (Brickman et al., 1981, p. 173). There are several theoretical and empirical attempts in the tradition of distributive justice research to summarize and sort rules and sub-rules of allocation. I started from some of these already existing “maps” (mainly referring to Bank, 2016; Brickman et al., 1981; Törnblom & Kazemi, 2015; Vietze, 2018; Voswinkel & Kocyba, 2008), which are summarized in Fig. 1, as sensitizing concepts for the analysis. In the course of the analysis, these concepts were adapted and extended for the Cuban case.

## Empirical Proceeding and Data

Based on the above-described assumptions regarding state-socialist egalitarian ideology, the initial overarching research question for the empirical analysis of the Cuban case was as follows: *Which (e)valuation patterns of distributive justice are prevalent in the institutional and discursive framework of Cuban state-socialism in different time periods?* To approach this question, I mainly referred to the interpretive analytics and methodological tools of the Sociology of Knowledge Approach to Discourse (Keller, 2006, 2013). With an extensive *discourse field exploration* based on *contextual literature*,^3^*exploratory interviews*,^4^ and *discourse fragments*^5^ I aimed at getting a comprehensive overview of documents dealing with distributive justice in the realm of work and welfare since 1959. An initial data corpus contained 429 documents from the period between 1959 and 2019 that I considered particularly informative regarding the above-mentioned research question.^6^ This data corpus was then successively reduced based on theoretical sampling procedures (particularly procedures of minimum and maximum variation, accompanied by constant comparisons between different discourse fragments) and the insights gained from the field exploration.

Further, in-depth analyses and coding procedures focussed on two periods of time, which I refer to as *the foundation of the socio-economic model in the early 1960s* and *the update of the socio-economic model in the 2010s*. Both periods are particularly revealing regarding (e)valuation patterns of distributive justice in the Cuban framework of institutions and political leaders’ views. The early 1960s can be understood as a period in which the revolutionary government created the foundations of a new “system” of distributive justice in Cuba (including important political debates and decisions regarding property, wages, and welfare). As discussed in the introduction, the update of the socio-economic model, which can be more or less ranged in the 2010s, is said to be one of the most—if not the most—substantial reform processes since the beginning of the revolution. Thus, analysing and contrasting these two periods allows, on the one hand, to grasp a broad range of possible (e)valuation patterns of distributive justice at two decisive points of time in Cuban history. On the other hand, it also allows, to a certain extent, to address possible configurations, i.e. continuities or changes of these patterns over time.

The orientation towards these two periods enabled the sampling of *key texts and passages*^7^ for the in-depth analysis of discourse fragments. The 108 textual documents that were retained can be roughly divided into two types: On the one hand, I analysed speeches and statements of *key actors* holding leading positions in the Cuban government (which, in the following, I will mainly refer to as *political leaders*). On the other hand, I also addressed institutionalized (e)valuation patterns in legal texts, policy- and action programmes, party resolutions and national statistics. Additionally, I consulted the contextual literature and exploratory interviews for the assessment of actual rewards, as will be explained below. For the reconstruction of (e)valuation patterns of distribution, I referred to methodological proposals of Grounded Theory (Corbin & Strauss, 2008),^8^ adapted for discourse analysis (see Keller, 2013). Codes were assigned to text passages that gave answers to the overarching questions *“Who should get what and why”* and *“Who gets what and why”*, using the above-described sensitizing concepts. Note that all documents were analysed in their original language. All translations in the presentation of the results in the next sections are my translations.

## Analysis

The following sections discuss the results obtained from the in-depth analysis of discourse fragments of the above-described periods: *The foundation of the socio-economic model in the early 1960s* and *the update of the socio-economic model in the 2010s*. The main focus of the analysis is placed on *ideational conceptions*, i.e. ideas of just rewards, targeted or desirable distribution outcomes, and justifications of (in)equalities. Nonetheless, some sections also include context descriptions and short assessments of actual rewards and distribution outcomes as they are depicted in the contextual literature.

### The Early 1960s: Founding the Socio-Economic Model

As much of the contextual literature on Cuban history describes, after the revolution had overthrown the Batista regime in 1959, the revolutionary government took control over major parts of the Cuban economy in the early 1960s. This included the nationalization of the country’s main means of production as well as the introduction of central planning and price controls. As different sources in the contextual literature further describe (e.g. Farber, 2011; Mesa-Lago, 1994; Mesa-Lago & Pérez-López, 2013; Yaffe, 2012), the opinions of the revolutionary leaders on *how* societal production should be organized and distributed were not always unanimous. Note, however, that the following subsections focus less on these disagreements within the so-called *great debate* (a topic that has been extensively covered in the literature) but more on describing (e)valuation patterns of distributive justice that prevailed during this period.^9^

### Overall Distribution: Minimum, Need and the Economic Battle

In the early 1960s, answers to the question of how a just distribution in the revolutionary society (i.e. on the macro-level) should look like, generally remain quite vague. One of the patterns that emerges in different discourse fragments is the objective to fulfil minimum standards of biological and basic needs, particularly of formerly less privileged Cubans.

[1] Now, we had to go and regulate it [the salary] again, so that the least, the less ‘fortunate’ let’s say, those with the lowest wages and the most difficult conditions, could ensure minimum conditions.^10^

As shown by this quote from the then-minister of industries Ernesto “Che” Guevara in 1962, the objective to reach *minimum distribution standards* is used to justify state intervention in the form of balancing or equalizing measures (which in some cases include, for example, waiver requests addressed to Cubans whose situation is perceived as better off or privileged). Another type of statement from this period refers to ideas of an *ideal future distribution* that goes beyond the goal to fulfil the needs of the less privileged. We find this objective to increase the overall level of need satisfaction, for example, in the *1963 Social Security Act*:

[2] The fundamental objective of the Socialist Society is to increasingly satisfy, in accordance with what the country’s economic development allows, the material and social needs of the workers.

Very often, this conception of an ideal future distribution is linked to the argument that to realize this *fundamental objective of socialist society* in the future, the top priority at present is to increase national production, productivity and efficiency. In many discourse fragments, economic development is emphasized as a key solution to a wide range of social problems, *,* e.g. unemployment, poor medical care, and malnutrition. Such problems are generally seen as results of the flaws of capitalism. In this context, economic production is also depicted as a part of the *fight* for Cuban sovereignty and against US imperialism:

[3] the year 1961 has been a year of important triumphs, not only in the way of the armed defence, in the sector of the armed defence of our sovereignty but also – and perhaps with as much importance as in the other aspect – of the vigilant defence, by means of the production, of our sovereignty.

The framing of economic production in military terms is a recurring pattern in various discourse fragments. Often, it serves to establish a rhetorical connection between the emphasis on economic development and the defence of the national territory. Indeed, in the course of the analysis, I identified the pattern of *(winning) the economic battle* as a central storyline^11^ in the Cuban leadership’s discourse on distribution and distributive justice. The term is an *in-vivo code*^12^ that is used in various statements of both the early 1960s and the period of the update of the socio-economic model. Although the *economic battle* does not embody a distributive justice rule per se, it is closely linked to issues of distributive justice and conceptions of just rewards. In the early 1960s, for example, the importance of winning the *economic battle* is frequently used as an appeal to the Cuban population—or certain parts of it—to make sacrifices in the present and to renounce present advantages in order to benefit from (greater) advantages in the future. Thus, this economistic or productivist pattern can even serve to justify equalizing measures, i.e. measures that follow the idea of equal just rewards and/or result in—more or less—equal actual rewards*.*

As pointed out by Mesa-Lago (1994) and Mesa-Lago and Pérez-López (2013), it is difficult to assess how these objectives of overall distribution materialized in actual rewards and (in)equalities in the early 1960s, particularly due to missing data. However, as is also noted by other sources in the contextual literature, overall poverty and income inequalities decreased, indicating an “impressive redistribution of income in the first four years of the Revolution” (Mesa-Lago, 1994, p. 41).

### The Prerequisite of Hard Work and the Conception of Rewards as Incentives

In the course of the analysis, two overarching patterns were found to be closely linked to the question of how to win the *economic battle* and to realize intended distribution goals: the *constraint* or *prerequisite of hard work* and the *conception of rewards as incentives.* Most of the discourse fragments that provide answers to the question of “who should get?” are, implicitly or explicitly, related to the category of the worker: in the revolutionary (and socialist) society, people are entitled to rewards because they are, were or will be workers who contribute to societal production. The fundamental importance of hard work of each and everyone is, so to speak, upstream to all distribution issues. This is also reflected in the conception of social security as it figures in the above-cited *1963 Social Security Act*. Beneficiaries are entitled to social protection because they, or at least their kin, are contributing to the revolution’s *economic battle*. As we will see in the next section, even social rewards that present a high degree of decommodification (such as rewards distributed through the rationing system), are not completely disconnected from the *prerequisite of hard work.*

In this context, the use of another sub-rule of need emerges, namely the rule of *functional needs.* This (non-self-referential) rule is expressed in the idea that workers’ needs should be fulfilled for them to be able to provide the hard work required to win the *economic battle*. The following reflections made by the then-prime minister Fidel Castro on the role of vacations serve as an example:

[4] It is in the interest of the country, of the country’s economy and the country’s development that the workers get their vacations. And we must insist on that aspect, too, and organize them properly, providing all the facilities. […] the annual rest must be organized; and this will always be useful to the working class, and will be useful to the country, and will be useful to our economy.

Another pattern that can be understood as a functional pattern is the general conception of rewards in the institutional and discursive framework of the early 1960s. According to the contextual literature, one issue among the above-mentioned disagreements between Cuban leaders in the early 1960s was the *form* of rewards to be distributed, i.e. whether rewards should be predominantly *monetary, moral,* or *social.* However, what all arguments seem to have in common, no matter which specific form of rewards they defend, is that they conceive of rewards first and foremost as *incentives [estimulós]*. This will be further examined in the next sections.

### Social Rewards—The Example of Supply Distribution: between Need, Equality, and Abnormality

In general, the contextual literature and the coded discourse fragments refer to two types of *social rewards* or *social incentives*. The first type is social policy benefits in the narrower sense, such as old age, maternity, or sickness pensions. Indeed, social policy benefits can also be perceived as belonging to the category of monetary rewards, and thus, as a variation of workers’ wages. The second and more “pure” type of social rewards is the free and/or subsidized goods and services that the revolutionary state started to provide to (parts of) the population in the early 1960s. The contextual literature generally highlights such goods and services provided in the areas of nutrition and everyday consumer goods, education, housing, health care, transportation, sports, culture, and communication. One may indeed argue that this second type of social rewards contains egalitarian elements, since, in their conception, they refer to the allocation rules of equality of treatment and/or equality of results, as well as biological and/ or basic needs. However, although many of these social rewards can be conceived of as universal and highly decommodifying rewards, they are seldom completely disconnected from the above-described prerequisite of hard work, i.e. the expectation for the beneficiaries to contribute to the *economic battle*.

Since the wide range of social rewards covering various areas cannot be discussed here, I will, in the following, focus on the example of *supply distribution* (which, in the contextual literature, is generally referred to as the *rationing system*). In March 1962, the revolutionary government passed the first law on the organization of a supply distribution system. This led, in 1963, to the introduction of supply booklets (called *Libreta de Abastecimiento,* in Cuba often simply referred to as the *Libreta)* recording the goods and quantities to be distributed to households.

[5] It is the Revolutionary Government’s duty to address this abnormal situation by organizing a form of distribution that results equitable [que resulte equitativa] and allows all zones of citizenship [a todas las zonas de la ciudadanía] equal access to everyday consumer goods [igual acceso a los artículos de consumo corriente], thus eliminating the distribution shortcomings that have arisen as a result of the situation described above.

As shown by this passage from the 1962 law, the conception of supply distribution refers to the allocation rules of equality of treatment as well as biological and basic needs. Interestingly, the form of distribution that should result from supply distribution is, in this context, not referred to as *outcome equality*, but as *equitable*. Furthermore, it shows that the target of achieving an equal (or equitable) distribution is not the only justification for the introduction of supply distribution. Another reasoning frame becomes clearer if we look at the paragraphs preceding the above-quoted passage. These paragraphs notably specify the *abnormal situation,* namely as a “situation of relative scarcity of certain items” that “has been used for anti-social and counterrevolutionary elements to speculate on some and encourage other campaigns aimed at promoting hoarding and encouraging consumer uncertainty”. Since the abnormal situation is attributed particularly to “the brutal economic siege led by the US imperialism against the national economy”, one can conceive of the introduction of supply distribution as part of the *economic battle*. Thus, both distributive justice and other patterns are present in the conception of supply distribution in the early 1960s. What we may note here in passing is that none of the analysed statements that justify the introduction of supply distribution regard it as a fundamental human right to an unconditional basic income.

Regarding *actual rewards* and *distribution outcomes* linked to supply distribution, Mesa-Lago (1994, p. 55) notes that between 1964 and 1966, “the extension of rationed items was an equalizer, but the rising prices of the growing black market had the opposite effect”. Further implications of supply distribution for actual rewards will be discussed in the section that presents the period of the update of the socio-economic model.

### Monetary Rewards—Wage Scales and Work Norms: Between Outcome Equality and Equity

As described by the contextual literature, in the early 1960s, the Cuban state had monopolized large parts of the economy and became the most important employer in the country: by 1963, about two-thirds of the Cuban working force were state employees (Mesa-Lago, 1994, p. 35). Integrating trade union action into governmental action had put a stop to collective bargaining in Cuba (see, for example, Hernández & Mesa-Lago, 1971; Farber, 2011). Gradually, the labour ministry introduced nationally unified wage scales and work norms, i.e. output standards and quotas defining production quantities and working time standards. This system of interconnected wage scales and work norms was supported even by political leaders who were said to be less in favour of monetary rewards. The following quote from Ernesto “Che” Guevara illustrates the perception of monetary rewards as a sort of necessary evil, a remnant of capitalism, which could, however, not be abolished immediately:

[6] Salary, that is, money, measures the different qualifications of all those who receive something for working. Money also measures the spirit of work of each of those who work in their different qualifications. Money is the only measure that can encompass everything, and in the era of the construction of socialism, when mercantile relations still exist, we have to work with money.

In its conception, the system of wage scales and work norms can be considered as a border case between the allocation rules of *equality of treatment, outcome equality* (notably in the form of collectively shared profits), and *equity.* The first two rules are expressed in the idea of *equal pay for equal work*, or, as it is also described by the revolutionary leaders, in the payment of wages independently of economic sectors and/or firms (and their varying profitability). The then-minister of labour Augusto Martínez-Sánchez phrased it as follows:

[7] [From now on], worker, you are going to earn according to your capacity, according to your work, and not depending on the profit of this or that enterprise, because … all of these enterprises are already yours, they belong to the people, and all workers must be equally evaluated.

This quote also shows that the system of wage scales and work norms contains the idea of *equity*, that is, the overarching rule of *distribution according to contribution*. In the following, I will briefly illustrate this what I call *(real)-socialist version* of the contribution rule by describing some of the categorizations and differentiations foreseen by the wage scales.^13^ The system included four overarching scales for four types of occupation, namely: agricultural workers, non-agricultural workers, administrative employees, technicians, and executive personnel. Each of the four occupational scales then further differentiated between seven or eight wage grades. These grades referred to degrees of complexity and precision of the job, i.e. the skills required by the worker. The basic wage of the workers was determined according to these four scales and corresponding grades. For wages of workers falling under the same occupational scale and wage grade, wages could vary according to four factors: labour conditions under which the work was performed, over- or nonfulfillment of work norms, overtime payments, and the so-called historical wages. The latter represented an additional payment to workers whose wages were lower with these new wage scales than in the pay schemes before the revolution. The over- or nonfulfillment of work norms resulted in bonuses and maluses, respectively. However, as noted by Hernández and Mesa-Lago (1971, p. 229), wage bonuses corresponded to only 50 percent of the overfulfillment. Moreover, the bonuses had a ceiling: in combination with the worker’s basic wage, they could not exceed the wage rate given to the next higher grade.

It is extremely difficult to assess *actual rewards* and resulting distribution outcomes linked to the introduction of wage scales and work norms. According to Mesa-Lago (1994, p. 36), it “helped to decrease salary differences (reducing particularly the income of the higher levels)”. Hernández and Mesa-Lago (1971, p. 231) note that they generally agree with “the thesis that the introduction of wage scales was a step forward in favour of egalitarianism because it reduced the differences in income that existed at the time in Cuba”. However, they point out that “certain qualifications are necessary” (ibid.): on the one hand, because of missing data (both from before and after 1959) and, on the other hand, because “extreme wage differences have continued throughout the period [of 1962–1966]” (ibid., p. 234). Regarding the latter issue, they further highlight persisting higher wages for technical and executive personnel that also enjoyed privileges, e.g. in the areas of housing, travel, and everyday consumer goods—which, under the Cuban conditions of general economic scarcity, may be highly valued. Incidentally, the analysis of the discourse fragments from this period showed how these privileges were justified—for instance, by highlighting their importance for winning the *economic battle*:

[8] So this technician, then, must be given certain facilities. And many times, it is true: a person can even receive an apartment having less need than another, because the industry, the country, needs that person.

Moreover, wage differences also continued to exist, e.g. in sectors of the economy in which wage scales and work norms were not applied, or only to a limited extent (e.g. in the agricultural sector, see Mesa-Lago, 1994). However, as the contextual literature describes (e.g. Hernández & Mesa-Lago, 1971; Mesa-Lago, 1994; Mesa-Lago & Pérez-López, 2013), in the second half of the 1960s, bonuses, overtime payments, and the so-called historical salary were gradually abolished. These sources also note that political leaders increasingly emphasized the use of *moral incentives* instead of material incentives*.* We will now take a closer look at the conception and (e)valuation patterns underlying this third type of reward.

### Moral Rewards—Socialist Emulation: Between Material Outcome Equality and Moral Equity

In the contextual literature, so-called *moral rewards*—mainly referred to as *moral incentives*—are generally linked to the political-economic philosophy of Ernesto “Che” Guevara and his followers. As described by the literature, moral incentives, in their positive form, could be individual (e.g. in the form of awards or medals for individual workers) or collective (e.g. in the form of awards for a firm or a working team). If applied as a substitute for monetary rewards, and in combination with the above-discussed social rewards, the conception of moral rewards may indeed contain the idea of *material outcome equality* and/or distribution according to *biological and/or basic needs*. However, in their basic conception, moral rewards are also closely linked to the idea of socialist emulation. This (real-)socialist form of competition was, at that time, expected to spur workers on to production and thus accelerate the construction of socialism. In this sense, moral rewards also express an aspect of the (real)-socialist version of the contribution rule, which includes both the idea of the measurability of work and the idea of the necessity of incentives:

[9] and we will only give the technical advice, the way to valuate, the way to measure emulation, so that the different types of work can achieve common measures that will later allow them to be compared with each other. At the same time, we have to establish incentives in emulation, moral incentives, such as how workers can see themselves individually or collectively in a workplace, as the best of the best.

Note that moral incentives can also be conceived of in a negative form, e.g. in the withdrawal of formerly received honours, or in public condemnation of workers who do not conform to the contribution rule. In this context, Farber (2011, pp. 145–150) notes a relationship between the increasing emphasis on moral incentives during the second half of the 1960s and, at the same time, reinforced control measures to strengthen labour discipline, sanctions, and forced labour.

### The 2010s: Updating the Socio-Economic Model

As described by the contextual literature, in the 2010s the Cuban state continued to have a monopoly on labour and the main means of production in the country. However, in the context of recurrent economic crises, observers note important changes in contemporary Cuban social and economic structures, such as increasing overall poverty and income inequalities. Authors also point to a gradual retraction of the state sector, particularly in its functions of main employer and property manager –accompanied by an increasing importance of other economic sectors (such as the so-called private, mixed, and cooperative sectors; many authors also highlight the importance of informal and illegal sectors in the contemporary Cuban economy). In this context, former president Raúl Castro^14^ had announced structural reforms already by the end of the 2000s. The resulting set of reforms is known as *the update of the socio-economic model* or (or simply *the update*) and was set out in the *Guidelines of the Social and Economic Policy of the Revolutionary Party* (2011). In 2016/2017 the Cuban Communist Party (PCC) approved three strategic documents containing changes of this reform agenda (Bye, 2020: 92–93; 103), among them an updated version of the *2011 guidelines*.^15^ In the following, I present some results of the in-depth discourse analysis for this period, focusing particularly on possible configurations of the categories and (e)valuation patterns carved out in the previous sections.

### Overall Distribution: The Economic Battle, Equality of Rights and Opportunities, and Contribution

The *seesaws* that the contextual literature notes for the entire history of the revolutionary government, its policies, and their rhetorical framing, seem to apply also to the framework of institutions and political leaders’ views in the 2010s. Nonetheless, many of the (e)valuation patterns described in the previous section for the 1960s, can be retrieved also in the 2010s, although with some modifications. The discursive storyline of the *economic battle* has continued until the recent past:

[10] The economic battle is today, more than ever, the main task and the centre of the ideological work of the cadres, because the sustainability and preservation of our social system depend on it.

[11] The economic battle remains the fundamental task and also the most complex one. It is the task that is most demanding of us all today, because it is the one our people expect the most from.

We have seen that in the early 1960s, the *economic battle* was often framed with the idea(l) of a better future for Cuban society. In the 2010s, the *economic battle* seems to have a threefold frame: it is depicted as fundamental to preserve past (social) achievements, meet the (peoples’) demands of the present and fight for a better future. Similar to the 1960s, the importance of ‘enlarging the cake’ and the consequent emphasis on economic constraints and requirements, including, above all, increases in efficiency and productivity, are put forward:

[12] Let us bear in mind the essential principle that in order to distribute wealth, it must first be created, and to do so, we must steadily raise efficiency and productivity.

The macrojustice rules of *minimum standards* and *biological and/or basic needs* are also present in several discourse fragments of the 2010s, where they are, in some cases, directly connected to the *economic battle*. In other cases, they are found as an ‘annex’ to the announcement of equity and/ or de-equalizing measures, oftentimes represented by the typified statement *that no one will be left without protection [desamparado]*. It seems, however, that there is a growing emphasis on individual responsibilities regarding the satisfaction of these needs. In the *Labour Code* adopted in 2013, we retrieve the pattern of a twofold prerequisite of work*:* by exercising their right and duty of work, citizens are expected to contribute to both societal development and the satisfaction of their individual needs. Interestingly, in this conception, it is through work incomes that contribution is made:

[13] work is a right and a social duty of the citizen and the income obtained from it is the fundamental way to contribute to the development of society and to the satisfaction of their personal and family needs.

Besides the ‘survival’ of these macrojustice patterns—albeit in slightly altered configurations –, another recurrent pattern identified for the 2010s is *equality of rights and opportunities,* which is frequently accompanied by criticism of *egalitarianism [igualitarismo]*. Note, however, that these patterns do not emerge in this period—especially *criticism of egalitarianism* can already be retrieved in discourse fragments of earlier decades. In the original *guidelines* of 2011, both patterns are coupled to a general conception of the equity rule:

[14] socialism means equality of rights and opportunities for all citizens, not egalitarianism. Work is simultaneously a right and a duty, a motivation for personal realization for each citizen, and should be rewarded according to its quantity and quality.

### Social and Monetary Rewards: Maintaining Revolutionary Conquests, Abolishing Gratuities, and a New Version of Equity?

Apart from the continuous emphasis on the *economic battle*, the prerequisite of (hard) work and contribution to society, some ruptures with the foundations of the framework of institutions and political leaders’ views, as we discussed them for the period of the early 1960s, can be observed. One example is the idea of moral rewards or incentives*,* which seems to have nearly disappeared in the 2010s. Regarding social rewards in general, both versions of the *guidelines* (2011; 2017), mention the objective for social policy to.

[15] [c]ontinue to preserve [2017: consolidate] the achievements of the Revolution, such as access to medical care, education, culture, sport, recreation, public peace, social security and protection through social assistance to those in need.

Note, however, that the phrasing of this objective is to preserve *access* to social rewards, but not *free access*—a conception of just rewards that various discourse fragments of the early 1960s highlight. If we look at the example of supply distribution, we can state that the *Libreta de Abestecimiento* has continued to exist—despite various announcements over the past decades to abolish this system of supply distribution. The aim of an “orderly and gradual elimination” of the supply booklets is also set out in the *guidelines* (2011; 2017)*.* Another passage of the *2011 guidelines* depicts the abolishment of such gratuities and subsidies as part of the *economic battle* (here represented by the goal to increase labour productivity, discipline, and motivation) and the organization of wages:

[16] it will be necessary to […] [i]ncrease labour productivity, raise the discipline and motivation level of wages and incentives by eliminating egalitarianism in the mechanisms of income distribution and redistribution. As part of this process, it will be necessary to eliminate undue gratuities and excessive subsidies.

In general, the necessity to realize the *socialist principle of distribution ‘from each according to capacity, to each according to work’* is a recurrent pattern in the period of the 2010s. It is often framed with the metaphor of an *inverted pyramid*. This metaphor is generally used to describe that qualification, work and income are not in the ‘right’ relation to each other, i.e. that professions requiring a high qualification are not rewarded with an accordingly high income. In turn, it is stated that people may have high incomes that are either non-work-related (e.g. because they receive remittances from abroad) or related to low skilled work (e.g. in the tourist sector, where tips can lead to high additional incomes). The *inverse pyramid* is perceived as a cause for low productivity, working motivation, and a brain drain within the Cuban economy, and thus as a threat to the *economic battle*. President Díaz-Canel quotes Raúl Castro’s description of this pyramid as follows:

[17] the unfair [injusta] inverted pyramid, in which with greater responsibility one receives lower pay and [in which] not all able citizens feel motivated to work legally, while at the same time discouraging the promotion to higher positions of the best and most qualified workers and cadres, some of whom emigrate to the non-state sector.

According to Cuban statistics (ONEI, 2019), in 2018, the state sector still employed around two-thirds of the Cuban labour force, despite relatively massive layoffs of state workers during the past decade (e.g. Bye, 2020). Here, wage scales and work norms are still in force. Although these have been subject to reforms and changes over the years, their principle composition in the 2010s remained similar to the early 1960s.^16^ Roughly described, wages in the scales that were in force until the end of 2020 consisted of a basic wage and additional payments for a wide range of possible criteria, such as working time, qualification, responsibility, seniority, individual, and team output.^17^ In the framework of the *update*, the Cuban government adopted several legal resolutions to regulate both basic wages and additional payments. In political debates, the latter have been discussed especially with regard to pay-for-performance systems in the state enterprise system, where *incentive bonuses [estímulos]* were (until the end of 2020) an important integral part of workers’ wages. However, in the case of non-achievement of (individual or team) working norms, bonuses could be subject to sanctions and thus to possible monthly fluctuations (in a range between zero to three times the basic wage of a worker).

In summary, we can state that in their ideational conception, state wage scales in the 2010s answered to the equity rule—we could say, to a *reconfiguration* of its real-socialist version as I described it for the 1960s. At the same time, this period is marked by strong criticism of low overall levels and differentiation of wages in the state sector, also voiced by the political leadership. Margarita González Fernández, minister of labour and social security, cites these problems in the context of wage increases in the public sector in 2019, insinuating the necessity of further wage reforms:

[18] this is a first step, it does not mean that it is the definitive solution, but it is a first step towards the solution of a problem of the current salary scale, regarding the very small differences between the different groups of complexity that did not stimulate to do better and drive.

This leads us to the assessments of *actual rewards* found in contextual literature for the period of the 2010s. Despite increasing nominal wages in the past decade, real wages in the state sector have been considered insufficient to cover daily living expenses in Cuba (Echevarría & Tejuca, 2017), and their purchasing power is said to have radically declined since the 1980s (e.g. Bye, 2020; Vidal, 2016). Anaya & Garcia (2018) estimated basic expenses for Cuban urban families of three, accounting for subsidies and supply distribution. They state that in 2016, an equivalent of two to three average state wages—or nine to ten minimum wages—of that year was necessary to cover such basic expenses. This stands in contrast to the above-mentioned high wages in other economic sectors, or non-work-related incomes (often in currencies offering a greater purchasing power than the Cuban Peso), and income privileges for certain groups (e.g. political and military elites). Additionally, the quality of and access to the so-called social rewards, e.g. of social security, health, and education, is said to have declined, especially since the crisis of the 1990s (e.g. Domínguez, 2017; Bye, 2020).

## Conclusion

The analysis of (e)valuation patterns of distributive justice in the Cuban framework of institutions and political leaders’ views, both in the early 1960s and the 2010s, has revealed a range of patterns that can indeed be classified as *egalitarian*. However, the results of the analysis allow us to make a few distinctions regarding the assumptions on *egalitarian state-socialist ideologies* that were addressed in the introduction of this article. By basing the analysis on the conceptual framework of the multiprinciple approach of justice, further (distributive justice) patterns and rules were found. In many cases, these are not incompatible with egalitarian ideology, but rather integrated into it, or, in some cases, used to criticize and update parts of it (without completely renouncing the overall ideology). Apart from patterns that are generally understood as expressions of an egalitarian ideology (in general, outcome equality and non-functional need rules), the analysis further pointed out patterns of equity (and its different sub-rules), functional needs, and other (functional and non-functional) allocation rules in the Cuban framework of institutions and political leaders’ views.

For instance, the analysis showed that functionalist allocation rules, often appearing in the context of economistic or productivist (e)valuation patterns, were already present in the ideas and actions of the Cuban revolutionary government in the early 1960s. They are not necessarily contradictory to egalitarian conceptions of overall distribution and just rewards but can in fact be “combined” with them—as, for example, the *economic battle* was used to justify equalizing just and/or actual rewards in the early 1960s. Regarding supposed changes in Cuban state-socialist ideology, the results suggest that many of the (e)valuation patterns of just rewards prevalent in the 2010s can be conceived of as reconfigurations of patterns of the early 1960s. For example, in the 2010s, minimum distribution standards and needs have not disappeared. However, corresponding patterns of the 1960s, such as moral incentives or the idea of equalizing measures to reach minimum standards, have become less important in the 2010s, or were partly replaced by other patterns, e.g. by the emphasis on equal opportunities, individual responsibilities, and the role of wages to fulfil basic needs. In this regard, one could indeed argue that *equality of rights and opportunities* has gained in importance with respect to *outcome equality* in the Cuban framework of institutions and political leaders’ views. However, as above-mentioned, it must be taken into account that the foundations for this change were laid way earlier (considering, for example, Fidel Castro’s early critiques of *egalitarianism* in the 1970s). As shown in the analysis, the equity rule (and its sub-rules) is emphasized in both periods—to justify both moral and material incentives in the early 1960s, and to criticize the *inverted pyramid* in the 2010s.

**Fig. 2:**
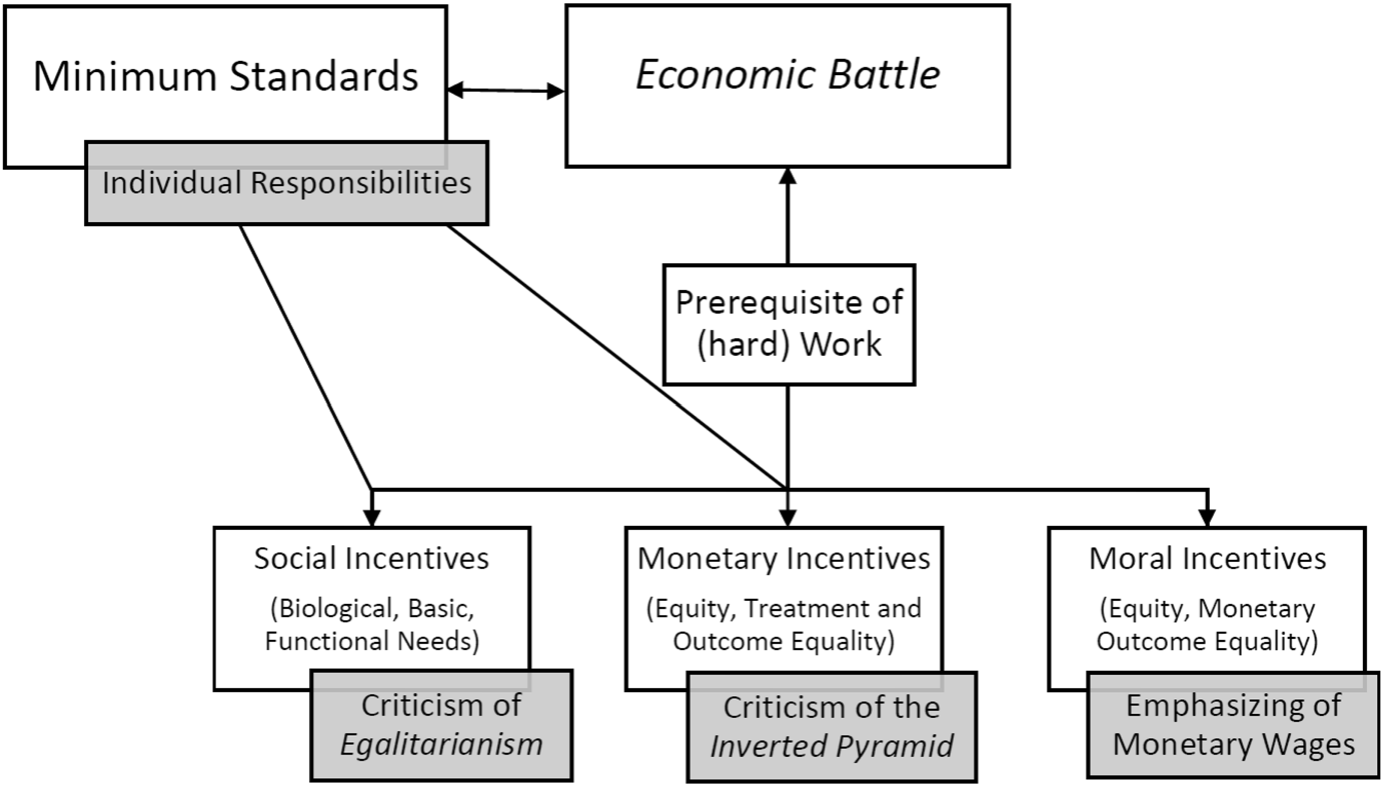
Basic (e)valuation patterns (white) and their updates (grey)

Please note in this context that with the above-described results, *changes over time* in the Cuban framework of institutions and political leaders’ views can only be cautiously addressed here. Since the results are based on a purely qualitative analysis focussed on two specific periods, they provide information mainly on the occurrence of (e)valuation patterns in these periods, and less so on changes in salience of these patterns over time (Fig. 2).

All in all, based on the results of the Cuban case, we can, in a more general manner, reject the assumption that *egalitarian state-socialist ideology* is merely based on (non-functionalistic and non-meritocratic) allocation rules of outcome equality and need. For many, this insight will be neither new nor surprising. Indeed, the presence of equity patterns in Cuban state-socialist ideology can even be traced back to Marxist theory. According to Marx (1973, pp. 24–25), the allocation rule “from each according to ability, to each according to needs” would be realized only at “a higher state of communist society”. Until then, socialist distribution would necessarily be based on work—and thus on some form of the equity rule and its sub-rules. Supposing that Cuba, like other real-socialist societies, has never reached this “higher state,” the emphasis on equity allocation rules does not seem surprising. Indeed, sub-rules of equity contain the promise of commensurability, i.e. the promise to provide more or less exact standards to measure work as a contribution to society, which is fundamental to the conception of planning economies. At the same time, the “flexibility of the original equity formulation” (Fischer, 2016, p. 467) allows for different interpretations of equity and its sub-rules, so that, as we have seen, the “socialist principle of distribution” may be configured and reconfigured for different contexts and time periods in the same country. Similarly, the prevalence of economistic and productivist patterns in state-socialist societies based on Marxist conceptions of socialism is not surprising either, considering that some parts of Marx’ economic theory defend, among other aspects, the primacy of the economy and labour value theory (see, for example, Heinrich, 2009; Streckeisen, 2018).

However, the assumptions on *egalitarian state-socialist ideologies*—as well as on (allegedly) opposite ideologies in (market-)capitalist societies –, which were addressed in the introduction, are still under discussion. The analysis of the Cuban case presented in this article may serve as a reminder that, “as equality may be conceptualized in a variety of ways, it is important to avoid miscommunication by being explicit about the specific (sub)type of equality at hand” (Törnblom & Kazemi, 2015, p. 30)—be this in state-socialist or market-capitalist contexts. Adopting such a stance may then also serve to revisit, for example, the conception of “communist variables” (based on cultural approaches) in comparative attitude research, as discussed in the introduction.

Moreover, the conceptual distinction of actual and just rewards defended in this article may serve as a reminder that any conception of a just distribution—also a so-called egalitarian or needs-based conception—is likely to be accompanied by *actual unequal outcomes*. The results of the analysis presented in this article showed some examples of how such inequalities are justified or criticized in both the early 1960s and the 2010s by referring to different (e)valuation patterns. Although actual rewards have only been addressed very briefly in this article, at least one observation for future Cuban-specific research can be made at this point: Studies that aim to examine individual attitudes towards distributive justice in Cuba should, besides considering (supposed) changes in ideology, account for a distribution of actual rewards in contemporary Cuba that is characterized by increasing (overall, sector-, and group-specific) inequalities. The defence or rejection of allocation rules and the conception of just rewards on an individual level may (highly) depend on (perceptions of) the distribution of actual rewards and/or their consistency with conceptions of just rewards.^18^

## Appendix

The quotes in the chapter *Analysis* were chosen according to their density of information and/or their potential represent further discourse fragments, i.e. to serve as typical or exemplary statements.

References to the quotes:

[1] Ernesto “Che” Guevara (30 April 1962): Speech at the Ministry of Industry’s Award Ceremony for Outstanding Workers. Source: http://www.cubadebate.cu/especiales/2018/06/14/ernesto-che-guevara-socialismo-es-productividad-mas-conciencia/. Accessed 14 June 2019.

[2] Law no. 1100 of 2 March 1963. Source: Finanzas al Día (1963). *Leyes del Gobierno Revolucionario de Cuba. Marzo, Abril y Mayo de 1963*. La Habana: Editorial Nacional de Cuba.

[3] Ernesto “Che” Guevara (6 January 1962): Speech at the General Assembly of Port Workers. Source: Guevara, E. C. (1977). *Escritos y Discursos 6*. La Habana: Editorial de Ciencias Sociales.

[4] Fidel Castro (28 November 1961): Speech at the XI Congress of the Cuban Trade Union Federation. Source: http://www.cuba.cu/gobierno/discursos/1961/esp/f281161e.html. Accessed 14 October 2019.

[5] Law no. 1015 of 12 March 1962. Source: Finanzas al Día (1962). *Leyes del Gobierno Revolucionario de Cuba. Marzo, Abril y Mayo de 1962.* La Habana: Editorial Nacional de Cuba.

[6] Ernesto “Che” Guevara (30 April 1962): Speech at the Ministry of Industry’s Award Ceremony for Outstanding Workers. Source: http://www.cubadebate.cu/especiales/2018/06/14/ernesto-che-guevara-socialismo-es-productividad-mas-conciencia/. Accessed 14 June 2019.

[7] Augusto Martínez Sánchez (16 May 1964): “A un mayor trabajo y una mayor calificación, un mayor salario", newspaper article in *Hoy*, 16 May 16 1964. Source: Hernández, R. E. & Mesa-Lago, C. (1971). Labor Organization and Wages. In C. Mesa-Lago (Ed.), *Revolutionary Change in Cuba.* Pittsburgh: University of Pittsburgh Press, 209–249.

[8] Fidel Castro (29 August 1966): Speech at the XII Congress of the Cuban Trade Union Federation. Source: http://www.fidelcastro.cu/es/discursos/discurso-pronunciado-en-la-clausura-del-xii-congreso-de-la-ctc-r. Accessed 30 October 2019.

[9] Ernesto “Che” Guevara (15 April 1962): Speech at the National Council of the Cuban Trade Union Federation. Source: Guevara, E. C. (1977): *Escritos y Discursos 6*. La Habana: Editorial de Ciencias Sociales.

[10] Raúl Castro (4 April 2010): Speech at the Congress of the Cuban Young Communist League. Source: http://www.cuba.cu/gobierno/rauldiscursos/2010/esp/r030410e.html. Accessed 28 October 2019.

[11] Miguel Díaz-Canel (23 March 2018): Speech at the Council of Ministers. Source: http://www.escambray.cu/2018/diaz-canel-la-batalla-economica-sigue-siendo-la-tarea-fundamental-y-tambien-la-mas-compleja/. Accessed 21 October 2019.

[12] Raúl Castro (22 February 2014): Speech at XX Congress of the Cuban Trade Union Federation. Source: http://www.cubadebate.cu/opinion/2014/02/22/discurso-de-raul-en-la-ctc-para-distribuir-riqueza-primero-hay-que-crearla. Accessed 28 October 2019.

[13] Law No. 116/2013 of 17 June 2014. Source: Gaceta Oficial de la República de Cuba, Minesterio de Justicia, Gaceta Oficial No. 29 Extraordinaria de 17 de junio de 2014: La Habana.

[14] Guidelines 2011: Lineamientos de la Política Económica y Social del Partido y la Revolución. Source: http://www.cubadebate.cu/noticias/2011/05/09/descargue-encubadebate-los-lineamientos-de-la-politica-economica-y-social-pdf. Accessed 5 September 2020.

[15] Guidelines 2017: Lineamientos de la Política Económica y Social del Partido y la Revolución para el período 2016–2021. Source: https://planipo-lis.iiep.unesco.org/sites/planipolis/files/ressources/ultimo_pdf_32.pdf. Accessed 5 September 2020.

[16] Guidelines 2011: Lineamientos de la Política Económica y Social del Partido y la Revolución. Source: http://www.cubadebate.cu/noticias/2011/05/09/descargue-encubadebate-los-lineamientos-de-la-politica-economica-y-social-pdf. Accessed 5 September 2020.

[17] Miguel Díaz-Canel (21 December 2019): Speech at the National Assembly. Source: http://www.granma.cu/discursos-de-diaz-canel/2019-12-21/diaz-canel-en-el-parlamento-cubano-unidos-hemos-vencido-unidos-venceremos. Accessed 25 October 2019.

[18] Margarita González Fernández (2 July 2019): Statement in the TV talk show *Mesa Redonda*, partially quoted on the related website (quote transcribed from the original video). Source: http://www.mesaredonda.cubadebate.cu/mesa-redonda/2019/07/02/diaz-canel-la-suma-de-todos-esos-esfuerzos-haga-el-milagro-de-la-prosperidad-sostenible-a-la-que-no-hemos-renunciado. Accessed 19 October 2019.

## Footnotes

1 The Cuban revolution is generally conceived of as a process, rather than a one-time event. Although this perspective may be debatable (see, for instance, Gropas, 2007), it will be adopted in this article for the sake of convenience.

2 These authors actually distinguish between consummatory and instrumental functions of justice.

3 I employ the term *contextual literature* for sources that I used for an introductory, informative analysis. These sources mainly consist of academic and popular literature on the history of the Cuban revolution and its socio-economic policies. Note, however, that the conceptual distinction between contextual literature and discourse fragments (see footnote 5) may, in the “practice” of discourse analysis, not always be clear, as contextual literature may also be part of the discourse that is analysed.

4 Five guided, half-structured interviews conducted with academics of the University of Havana in January/ February 2018. Note that, like the contextual literature, the interviews may also be considered as discourse fragments.

5 A discourse fragment is a “[s]tatement-event (utterance) in which discourses are more or less comprehensively actualized (e.g. a text); [it is the] principal database for analysis” (Keller, 2013, p. 73).

6 This somewhat less systematic approach was adopted particularly in view of the long time span (and the resulting multitude of corresponding documents) considered at beginning of the analysis. Thus, to a certain extent, sampling procedures rely on the researcher’s (subjective) considerations and interpretations. Yet, as explained, these are based on a thorough exploration of the discursive field, and oriented towards the guidelines on corpus building and the selection of data provided by Keller (2013, pp. 95–101).

7 Key texts and passages can be understood here as particularly dense, relevant and informative texts with respect to the research questions, and as containing typical or exemplary statements. They were selected according to their density of information and/or their potential to represent further discourse fragments.

8 That is, the ideas of open, axial und selective coding, as well as memos and commentaries.

9 The reader may notice that Ernesto "Che" Guevara and Fidel Castro are quoted relatively frequently in this section. This should not give the impression that there was an ideologically homogeneous leadership at the time that unanimously espoused Guevara's or Castro's ideas. Yet, following Mesa-Lago (1971, 1994), Guevara and Fidel Castro count as "the two most important and productive leaders of the revolution" (Mesa-Lago, 1971: 536), and the "guevarist-castrist model" (Mesa-Lago, 1994: 127) prevailed in the second half of the 1960s. Against this background, the prioritisation of their views at this point seems justified.

10 References to the quotes are provided in the *Appendix*.

11 According to Keller (2013, p. 73) a storyline is a “common thread of a discourse by means of which the different constituents of the interpretative repertoire are interconnected”.

12 Corbin & Strauss (2008, p. 65) define an in-vivo code as a term that comes out directly from the data, “rather than being named by the analyst”.

13 As original data on this matter is difficult to find, I mainly refer here to the data of Hernández & Mesa-Lago (1971), who, based on different sources, provide a comprehensive overview of the wage scale system in the mid-1960s.

14 Raúl Castro became acting president of the Republic of Cuba in 2006 to represent his sick brother Fidel and was officially appointed president in 2008.

15 New reform packages were adopted again at the end of 2020/beginning of 2021, in the framework of establishing a *new monetary order [ordenamiento monetario]* and resolutions of the eighth congress of the PCC in April 2021. However, at the time of writing, these changes could not yet be included in the analysis.

16 Note that some substantial changes were made to the wage scales in 2020/21, which, as explained in footnote 15, could not be included in the analysis.

17 See, for example, the overview provided by Galtés (2016).

18 Of course, this suggestion may also apply to the international attitude research mentioned in the introduction. On an empirical level this could mean, e.g. precisely differentiating between items on just rewards and items on actual rewards in questionnaires—which is not always the case in existing questionnaires. Janmaat’s (2013) literature review with its conceptual distinction in perceptions, beliefs and judgements of *fair* and *existing* inequalities offers interesting insights in this regard.
